# Compressive Sensing Spectroscopy Using a Residual Convolutional Neural Network

**DOI:** 10.3390/s20030594

**Published:** 2020-01-21

**Authors:** Cheolsun Kim, Dongju Park, Heung-No Lee

**Affiliations:** School of Electrical Engineering and Computer Science, Gwangju Institute of Science and Technology, Gwangju 61005, Korea; csk0315@gist.ac.kr (C.K.); toriving@gist.ac.kr (D.P.)

**Keywords:** spectroscopy, compressed sensing, deep learning, inverse problems, sparse recovery, dictionary learning

## Abstract

Compressive sensing (CS) spectroscopy is well known for developing a compact spectrometer which consists of two parts: compressively measuring an input spectrum and recovering the spectrum using reconstruction techniques. Our goal here is to propose a novel residual convolutional neural network (ResCNN) for reconstructing the spectrum from the compressed measurements. The proposed ResCNN comprises learnable layers and a residual connection between the input and the output of these learnable layers. The ResCNN is trained using both synthetic and measured spectral datasets. The results demonstrate that ResCNN shows better spectral recovery performance in terms of average root mean squared errors (RMSEs) and peak signal to noise ratios (PSNRs) than existing approaches such as the sparse recovery methods and the spectral recovery using CNN. Unlike sparse recovery methods, ResCNN does not require *a priori* knowledge of a sparsifying basis nor prior information on the spectral features of the dataset. Moreover, ResCNN produces stable reconstructions under noisy conditions. Finally, ResCNN is converged faster than CNN.

## 1. Introduction

There has been considerable interest in producing compact spectrometers having a high spectral resolution, wide working range, and short measuring time. Such a spectrometer can be used in a broad range of fields such as remote sensing [1], forensics [2], and medical applications [3]. Spectrometers that exploit advanced signal-processing methods are promising candidates. The compressive sensing (CS) [4,5] framework makes it possible for a spectrometer to improve its spectral resolution while retaining its compact size. CS spectroscopy comprises two parts: Capturing a spectrum with a small number of compressed measurements and reconstructing the spectrum from the compressed measurements using reconstruction techniques.

To date, for effective signal recovery in CS spectroscopy, three requirements should be satisfied. First, the spectrum should be a sparse signal or capable of sparse representation on a certain basis. Second, the sensing patterns of optical structures should be designed to have a small mutual coherence [6]. Third, appropriate reconstruction algorithms are required. Note that several sparsifying bases have been used in CS spectroscopy such as a family of orthogonal Daubechies wavelets [7], a Gaussian line shape matrix [8,9], and a learned dictionary [10]. Furthermore, numerous optical structures have been proposed to attain the necessary small mutual coherence for sensing patterns such as thin-film filters [11,12], a liquid crystal phase retarder [13], Fabry–Perot filters [7,14], and photonic crystal slabs [15,16]. As algorithms for reconstructing the original signal, two types of basic reconstruction techniques have been developed: greedy iterative algorithms [17,18] and convex relaxation [19,20]. In CS spectroscopy, the reconstruction algorithms have been used with a sparsity constraint. Additionally, a non-negativity constraint is used in Reference [16,21]. Combining these three considerations, CS spectrometers have shown stable performance for light-emitting diodes (LEDs) and monochromatic lights.

Since not all signals can be represented as sparse on a fixed basis, prior information on structural features of the spectral dataset is therefore required to generate a best-fit sparsifying basis. Furthermore, a high computational cost is required for reconstruction techniques. Recently, deep learning [22] has been emerging as a promising alternative framework for reconstructing the original signal from the compressed measurements.

Mousavi et al. [23] was the first study on image recovery from structured measurements using deep learning. Moreover, a deep-learning framework for inverse problems has been applied in biomedical imaging for imaging through scattering media [24], magnetic resonance imaging [25,26], and X-ray computed tomography [27]. Kim et al. [28] reported the first attempt to use deep learning in CS spectroscopy. They trained a convolutional neural network (CNN) to output the reconstructed signal from the network. From here on the network reported by Kim et al. will be referred to as CNN.

Unlike CNN [28] in which learnable layers were simply stacked and trained to directly reconstruct the original spectrum, we make a residual connection [29] between the input and output of CNN and train the network to reconstruct the original spectrum by referring the input of the network. As a result, the network learns residuals between the input of the network and the original spectrum. It has been reported that it is easier to train a network when using residual connections than to train a plain network that was simply stacked with learnable layers [25,29]. Lee et al. [25] analyzed the topological structure of magnetic resonance (MR) images and the residuals of MR images. They showed that the residuals possessed a simpler topological structure, thus making learning residuals easier than learning the original MR images. In addition, He et al. [29] demonstrated with empirical results that the residual networks are easy to optimize and they achieved improvements in image-recognition tasks. From these works, we gain insights such that adding residual connections to CNN would improve the spectral reconstruction performance in CS spectroscopy.

In this paper, we aim to propose a novel residual convolutional neural network (ResCNN) for recovering an input spectrum from the compressed sensing measurements in CS spectroscopy. The novelty lies in the proposed ResCNN structure, with a moderate depth of learnable layers and a single residual connection, which provides the desired spectral reconstruction performance. The desired performance here means that the proposed ResCNN offers a performance which is better than that of CNNs as well as that of CS reconstruction with its sparsifying base known. In CS reconstruction, the prior knowledge of a fixed sparsifying basis is useful and offers good sparse representation results. However, in general it is a difficult problem to identify a sparsifying basis for various kinds of spectra and apply the identified basis to have the recovery performance improved. In this regard, it is an important advance to find a simple ResCNN which offers good enough performance. It is also worth to note that the proposed ResCNN is tested with the array type CS spectroscopy, discussed in Section 2, which we have designed with an array of multilayer thin-film filters.

The previous works on CS spectroscopy [7,11,13,14,16] have shown decent reconstruction performance but on limited simple sources such as LEDs and monochromatic lights. Using ResCNNs, we are now able to reconstruct more complex spectra, such as spectra with multiplicity of peaks mixed with a gradual rise-and-fall. 

The remainder of this paper is organized as follows. In Section 2, we model the optical structure which is used for CS spectroscopy. In Section 3, we describe the system of CS spectroscopy and the proposed ResCNN. In Section 4, simulated experiments are described. Section 5 presents the results of experiments. In Section 6, we discuss the results. Finally, we conclude this paper in Section 7.

## 2. Optical Structure

Numerous optical structures have been proposed for CS spectroscopy. It has been reported that CS spectrometers, which have various spectral features in the transmission spectrum, show high spectral-resolving performance [16]. In this work, we used thin-film filters to model CS spectrometers. Thin-film filters demonstrate a variety of spectral features depending on the materials used, the number of layers, and the thicknesses of the layers. Once the structure of thin-film is determined, a transmission value at a given wavelength λ is defined as follows [30]:

(1) Tλ=1−12ρTEλ2+ρTMλ2,

where $\rho_{TE}\left(\lambda \right)$ and $\rho_{TM}\left(\lambda \right)$ are amplitude reflection coefficients. The coefficients represent the fraction of the power reflected by a multilayer thin-film in the transverse electric (TE) and transverse magnetic (TM) modes of an incident light, respectively. We summarized recursive processes for calculating amplitude reflection coefficients in Algorithm 1 [11,12,31].
**Algorithm 1:** Recursive processes for amplitude reflection coefficients.
**Input:**λ
Structure parameters: $\theta_{1},$$n=\left\{n_{1},n_{2},\cdots ,n_{l}\right\},$$d=\left\{d_{2},d_{3},\cdots ,d_{l}\right\}.$
**Step 1:**Calculate $\theta_{k},$$\beta_{k},$ and $N_{k}$ using structure parameters.
$\theta_{k}=\sin^{-}\left(\frac{n_{k-1}}{n_{k}}\sin\theta_{k-1}\right),$ for *k* = 2, 3, ..., *l*. $\beta_{k}=2\pi \cos\left(\theta_{k}\right)n_{k}d_{k}/\lambda ,$ for *k* = 2, 3, ..., *l*. $N_{k}=\left\{\begin{array}{cc} n_{k}/\cos\theta_{k} & \begin{array}{cc} for & TE \end{array}\\ n_{k}\cos\theta_{k} & \begin{array}{cc} for & TM \end{array} \end{array}\right.,$ for *k* = 2, 3, ..., *l*.**Step 2:**Obtain $\eta_{2}$ by setting $\eta_{l}=N_{l}.$

For *k* = *l*-1 to 2 $\eta_{k}=N_{k}\frac{\eta_{k+1}\cos\beta_{k}+jN_{k}\sin\beta_{k}}{N_{k}\cos\beta_{k}+j\eta_{k+1}\sin\beta_{k}}.$
**Step 3:**Compute $\rho =\left(N_{1}-\eta_{2}\right)/\left(N_{1}+\eta_{2}\right).$
**Output:**ρ

Here, $\theta_{k}$ is the angle of an incident light passing from *k*^th^ to *k*+1^th^ layer. The refractive index of *k*^th^ layer is denoted as $n_{k}.$ $d_{k}$ denotes the thickness of the *k*^th^ layer. Given a wavelength vector $\lambda =(\lambda_{1}\;\lambda_{2}\;\ldots \;\lambda_{N})\in {\mathbb{R}}^{1\times N}$ in the range of interest, i.e., $\lambda_{\max}-\lambda_{\min}.$ Let $\Delta \lambda =\frac{\lambda_{\max}-\lambda_{\max}}{N}.$ Then, evaluating the function at the integer multiple of Δλ, i.e., $T(\lambda =\lambda_{\min}+n\Delta \lambda )$ for n=0,1,⋯,N−1, we obtain the vector of transmission spectrum $T_{m}\in {\mathbb{R}}^{1\times N}$ for the wavelength range. Then, the sensing pattern matrix of optical structures $T\in {\mathbb{R}}^{M\times N}$ is obtained by repeating the calculation of $T_{m}$ for m=1,2,⋯,M.

We have used SiNx and SiO_2_ for high- and low-refractive index materials, respectively. We numerically generated thin-film filters by alternately stacking high- and low- refractive index materials, changing the number of layers, and varying the thickness of each layer. The number of layers in each filter is in the interval of (19, 24), and the thickness (nm) of each layer is in the interval of (50, 300). Initially, we randomly generated reference filters and compute the mutual coherence among the filters. Then, new filters were generated by changing thicknesses of the layers and the mutual coherence of the filters is compared to the mutual coherence of reference filters. Filters with a smaller mutual coherence then became the new reference filters. This process is repeated until reasonable reference filters with the required small mutual coherence are obtained.

**Figure 1:**
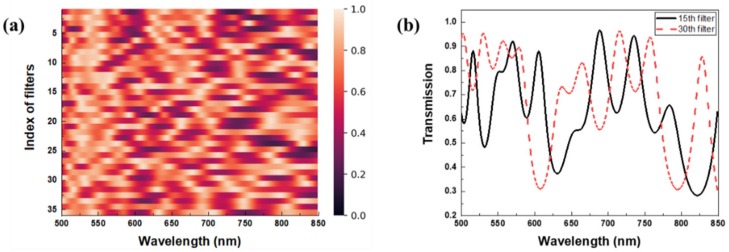
(**a**) Heatmap of the sensing matrix: each row represents the transmission spectrum of the designed thin-film filter. (**b**) Two transmission spectra corresponding to the 15th and 30th rows in the sensing matrix.

Figure 1 shows the heatmap for the transmission spectra of the reference filters and two selected transmission spectra. In Figure 1a, each of the transmission spectra shows a unique sensing pattern because of the iterative modeling process of the reference filters based on mutual coherence. Figure 1b shows two transmission spectra that correspond to the 15th and 30th rows in the heatmap of reference filters. The transmission spectrum reveals a deep spectral modulation depth and various features such as broadband backgrounds, multiple peaks with a small full width at half maximums (FWHMs), and irregular fluctuations.

## 3. Compressive Sensing (CS) Spectrometers Using the Proposed Residual Convolutional Neural Network (ResCNN)

### 3.1. CS Spectrometers

In CS spectroscopy, the measurement column vector $y\in {\mathbb{R}}^{M\times 1}$ is represented using the following relation:

(2) y=Tx,

where $x\in {\mathbb{R}}^{N\times 1}$ is the spectrum column vector of incident light and $T\in {\mathbb{R}}^{M\times N}$ is the sensing matrix of the optical structure. Each row of T represents a transmission spectrum. Because the length of the measurement vector is smaller than the length of the spectrum vector (*M* < *N*), the system is underdetermined. Conventionally, if x is a sparse signal or can be sparsely represented in a certain basis, i.e., x=Φs, reconstruction algorithms can determine a unique sparse solution $\hat{S}$ from the following optimization problem:

(3) $$\underset{s}{\min}{\left\|T\Phi s-y\right\|}_{2}^{2}+\tau {\left\|s\right\|}_{1},$$

where $\Phi \in {\mathbb{R}}^{N\times N}$ is a sparsifying basis and τ is a regularization parameter. Here, s is a sparse signal whose components are zero except for a small number of non-zero components. Then, the recovered spectrum $\hat{x}$ is $\Phi \hat{s}$. In this paper, we refer to the methods of solving the optimization problem using Equation (3) as sparse recovery.

Typically, except for narrow-band spectra, a spectrum is not a sparse signal, and a fixed sparsifying basis cannot transform all spectra into sparse signals. Clearly, the use of a fixed basis may lead the sparse recovery to struggle, as no fixed basis will transform every signal into a sparse signal. In addition, the sparse recovery is time-consuming and takes a high computational cost.

**Figure 2:**
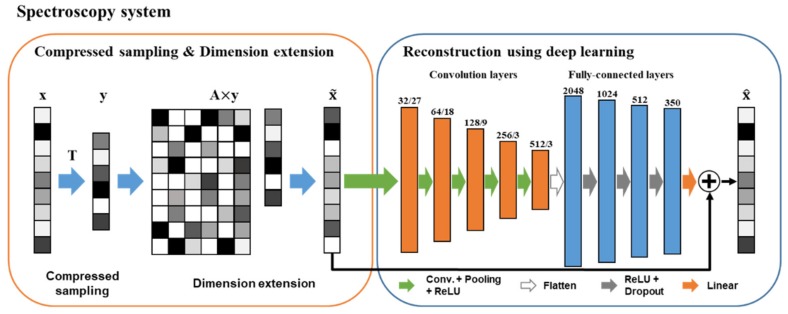
Overview of compressive sensing (CS) spectrosocopy system including the proposed residual convolutional neural network (ResCNN): An input spectrum is compressively sampled by the sensing matrix, and the dimension of measurements is extended by the transform matrix. ResCNN is trained to recover the original spectrum from the extented measurements.

Our goal is to overcome the limitations of the sparse recovery in CS spectroscopy and recover various kinds of spectra using ResCNN. Figure 2 shows the schematic of the CS spectroscopy system using ResCNN. This system consists of two parts: compressive sampling and dimension extension, and the reconstruction using ResCNN. In the compressive sampling and dimension extension, the measurement vector **y** is obtained from Equation (1), which then transforms into $\tilde{x}\in {\mathbb{R}}^{N\times 1}$ using a linear transformation. A transform matrix $A\in {\mathbb{R}}^{N\times M}$ extends the *M* dimension of **y** to *N* dimension of $\tilde{x}$, where $\tilde{x}$ is a representative spectrum corresponding to x. We used $\tilde{x}$ as the input for the reconstruction. ResCNN learnt a non-linear mapping between $\tilde{x}$ and x, and afforded a reconstructed spectrum $\hat{x}\in {\mathbb{R}}^{N\times 1}$. The dimension extension by the transform matrix was used to make it easier for ResCNN to extract features and reconstruct spectra from the non-linear mapping.

### 3.2. The Proposed ResCNN

As depicted in Figure 2, ResCNN comprises nine learnable layers, five of which are convolution layers, four are fully-connected layers, and one is a residual connection. Convolution layers are used for the feature extraction in the non-linear mapping between $\tilde{x}$ and x. Fully-connected layers are used for the spectra reconstruction. Each of the convolution layers has a set of one-dimensional learnable kernels with specific window sizes. The number of kernels and the window sizes are indicated in Figure 2. After every convolutional layer, the rectified linear unit (ReLU) is used as an activation function, and the subsampling is then applied. We use non-overlapping max-pooling to down-sample the output of the activation function. We stack the convolutional layer, the ReLU, and the subsampling five times. The output of the last subsampling is flattened and then fed into the subsequent four fully-connected layers. The first three layers are followed by the ReLU and dropout in sequence. The dropout is introduced to reduce the overfitting of ResCNN. The output of the last fully-connected layer is fed into a linear activation function. The number of units in each of the fully-connected layers is noted in Figure 2. Unlike CNN [28] in which learnable layers are simply stacked, we make the residual connection that the representative spectrum $\tilde{x}$ and the output of the linear activation function are added up to the reconstructed spectrum $\hat{x}$. Consequently, $\hat{x}$ is trained to become **x**. Given training data ${\left\{x_{t}{}^{i}\right\}}_{i=1}^{k}$, we train ResCNN to minimize a loss function *L*. We use the mean squared error between the original $x_{t}$ and recovered ${\hat{x}}_{t}$ as the loss function:

(4) $$L=\frac{1}{k}\sum_{i=1}^{k}{\left\|x_{t}{}^{i}-{\hat{x}}_{t}{}^{i}\right\|}_{2}^{2}.$$

The non-linear mapping that $\tilde{x}$ becomes **x** can be defined as $H(\tilde{x})=x$. Because of the residual connection in ResCNN, $H(\tilde{x})$ can be rewritten as $H(\tilde{x})=F(\tilde{x})+\tilde{x}$, where $F(\tilde{x})$ is the mapping of the learnable layers. The representative spectrum $\tilde{x}$ is referenced by the residual connection, and then,$F(\tilde{x})=H(\tilde{x})-\tilde{x}$. In particular, the mapping of $F(\tilde{x})$ is called a residual mapping; therefore, the learnable layers learn the residual of **x** and $\tilde{x}$.

**Figure 3:**
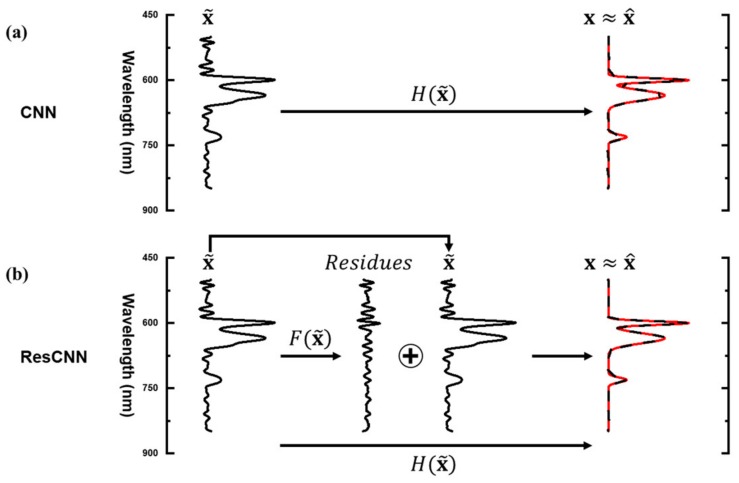
Descriptions of the spectrum recovery process: (**a**) convolutional neural network (CNN), (**b**) ResCNN.

The previous researches [25,29] have used numerous residual connections in very deep neural networks in order to make networks converge faster by avoiding vanishing gradient problems. We use one residual connection between input and output of the moderate depth network. Figure 3 depicts the manner in which a spectrum is recovered in CNN and ResCNN. The learnable layers of CNN directly reconstruct the spectrum from the representative spectrum $\tilde{x}$. Alternatively, ResCNN reconstructs the spectrum by passing the representative spectrum $\tilde{x}$ through the residual connection shown in Figure 3b. Consequently, the learnable layers of ResCNN learn to reconstruct residuals.

## 4. Simulated Experiments

We reconstructed 350 spectral bands (*N* = 350) using 36 thin-film filters (*M* = 36) whose sensing patterns have a spacing of 1 nm for wavelengths from 500 to 850 nm. We determined the sensing matrix **T**, assuming that the incident light falls onto the filters with normal incidence. As the transform matrix A, we used the Moore–Penrose inverse of the sensing matrix **T**, i.e., $A=T^{T}{\left(TT^{T}\right)}^{-1}$.

### 4.1. Spectral Datasets

**Figure 4:**
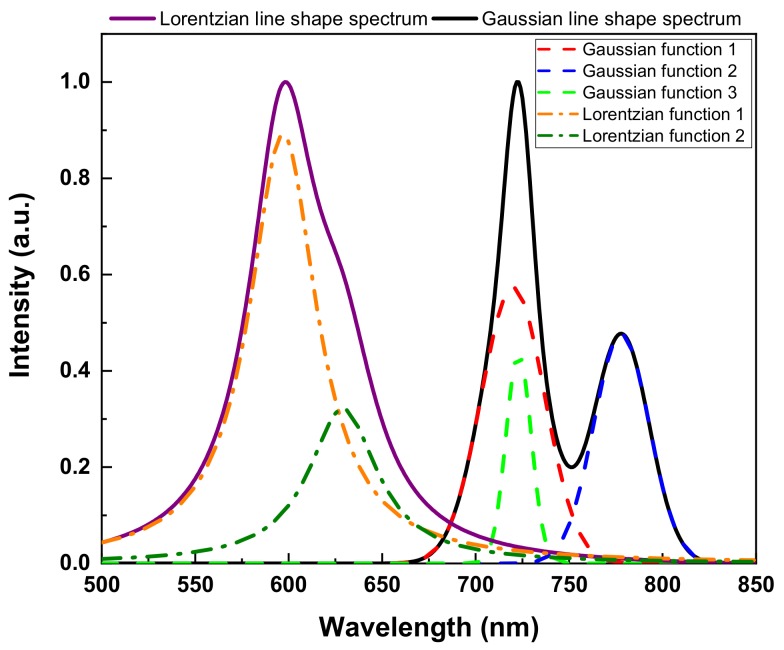
Examples of two synthetic spectra: the solid purple line is composed of two Lorentzian distribution functions (dash-dotted orange and olive lines), and the solid black line is composed of three Gaussian distribution functions (dashed red, blue, and green lines).

To evaluate the performance of ResCNN, we used two synthetic spectral datasets and two measured spectral datasets. The first synthetic dataset is composed of Gaussian distribution functions while the other is composed of Lorentzian distribution functions. These two synthetic datasets were selected as generally these types of functions are used to represent spectral line shapes. As shown in Figure 4, component functions are added to produce the spectra. We generated 12,000 spectra for each dataset. For each spectrum, the number of component functions was generated using a geometric distribution with the probability parameter p set to 0.3. We added one to the number of component functions to prevent the number of component functions from becoming zero. Then, we randomly set a location, a height, and an FWHM of each peak. To set a peak location (nm), an integer number was randomly selected from a uniform distribution with the interval (500, 849). A random number from a uniform distribution in the interval (0, 1) was used for the height. An integer number for an FWHM (nm) was randomly drawn from a uniform distribution with the interval (2, 50). Finally, all of the component functions were summed to generate the spectrum. The height of each generated spectrum was normalized such that it was mapped from zero to one.

As measured datasets, we used the US Geological Survey (USGS) spectral library version 7 [32], and the glossy Munsell colors spectral dataset [33]. The USGS spectral library provides reflectance spectra for artificial materials, coatings, liquids, minerals, organic compounds, soil mixtures, and vegetation. We discarded any spectrum that has missing spectral bands. Then, we extracted the spectrum in the wavelength range of interest (500 to 849 nm) from the wavelength range of the original spectrum (350 to 2500 nm). The measured wavelength range for the glossy Munsell colors spectral dataset, which contains the reflectance spectra of the glossy Munsell color chips, was 380 to 780 nm. The wavelength range of the original spectrum was different from the wavelength range of interest. We decided to use the wavelength range from 400 to 749 nm to ensure each spectrum was set to 350 spectral bands. This selection of wavelengths is reasonable because the wavelengths were located in the center of the wavelength range of the original spectrum, and showed different spectral features with respect to each spectrum. In addition, our aim was to show the reconstruction performance with respect to various kinds of spectra. Finally, each spectrum was normalized such that the height varies from 0 to 1. Overall, 1473 spectra from USGS spectral dataset and 1600 spectra from Munsell color spectral dataset were used for our simulated experiments. Table 1 lists the details of each of the spectral datasets.

### 4.2. Data Preprocessing and Training

Given the sensing matrix, the spectral data are compressively sampled as the measurement vector **y** shown in Equation (1), and then transformed into the representative spectrum $\tilde{x}$ by multiplying the transform matrix **A** and **y**.

In each spectral dataset, the number of training, validation, and test spectra are randomly assigned using a ratio of 4:1:1 for the synthetic and measured data sets, respectively. The validation spectra are used for estimating the number of epochs and tuning the hyper-parameters. To train ResCNN, we used the Adam optimizer [34] implemented in Tensorflow with the batch size of 16 and 250 epochs. The experiments were conducted on an NVIDIA GeForce RTX 2060 graphics processing unit (GPU). Training the architecture can be done in half an hour for each dataset.

### 4.3. Sparsifying Bases for Spare Recovery

Using sparse recovery, we evaluated the performance of conventional CS reconstructions to benchmark the performance of ResCNN. As shown in Table 1, the spectra for both the synthetic and measured datasets are dense spectra. Therefore, we must transform the spectra into sparse signals to solve Equation (3). In this section, we considered methods to make a sparsifying basis Φ.

**Figure 5:**
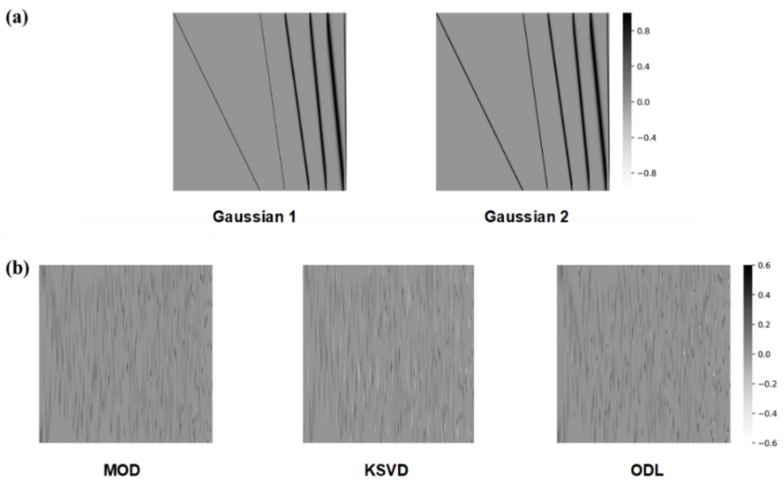
Heatmap images of sparsifying bases that were used in simulated experiments: (**a**) Gaussian line shape matrices, (**b**) the learned dictionaries which are from the Gaussian training dataset.

First, we considered a Gaussian line shape matrix as a sparsifying basis. Each column of the matrix comprises a Gaussian distribution function whose length is *N*. A collection of *N* Gaussian functions works as a sparsifying basis $\Phi \in {\mathbb{R}}^{N\times N}$. We generate two Gaussian line shape matrices. Figure 5 a shows the heatmap images for two Gaussian line shape matrices. Seven different FWHMs are used to generate the Gaussian distributions. Given an FWHM, Gaussian distributions are generated by shifting the peak location using uniform spacing. To create a small dissimilarity between the two Gaussian line shape matrices, two of the seven FWHMs in Gaussian 1 were replaced with other FWHMs, thus producing Gaussian 2, as shown in Figure 5a.

Second, a learned dictionary [35,36,37,38] is used as a sparsifying basis. Given a training dataset ${\left\{x_{t}{}^{i}\right\}}_{i=1}^{k}$, we can derive a learned dictionary Φ that sparsely represents the training data $x_{t}$ by solving the following optimization problem, known as the dictionary learning problem:

(5) $$\underset{\Phi ,s_{t}{}^{1},\ldots ,s_{t}{}^{k}}{\min}\sum_{i=1}^{k}{\left\|x_{t}{}^{i}-\Phi s_{t}{}^{i}\right\|}_{2}^{2}+\tau {\left\|s_{t}{}^{i}\right\|}_{1},$$

where τ is a regularization parameter and $s_{t}{}^{i}$ is *i*th sparse signal over the training dataset. By fixing an initial guess for the dictionary Φ in Equation (5), we obtain a solution for the sparse signals ${\left\{s_{t}{}^{i}\right\}}_{i=1}^{k}$. The dictionary is then updated by solving Equation (5) using the sparse signals obtained. This process is iteratively repeated until convergence is reached and we derive the learned dictionary. We used three dictionary learning methods: method of optimal directions (MOD) [36], K-SVD [37], and online dictionary learning (ODL) [38]. The learned dictionaries are generated for each of the training datasets, and the reconstruction performances are evaluated for each test dataset. Figure 5b shows learned dictionaries identified using the Gaussian training dataset. The learned dictionaries clearly depend on the dictionary-learning methods used. Nevertheless, each column of the dictionaries shows a learned spectral feature from the training dataset.

## 5. Results

To demonstrate the ability of ResCNN to reconstruct spectra, we evaluated its performance using three different datasets: Synthetic datasets, noisy synthetic datasets, and measured datasets. We used the same hyper-parameters of ResCNN for each of these datasets. Moreover, we adopted *l1_ls* [39] as the fixed reconstruction algorithm in the sparse recovery. We compared the recovered signal with the original signal by calculating the root mean squared error (RMSE) and the peak signal to noise ratio (PSNR). In addition, the performance of five conventional sparse recovery methods, described in Section 4.3 and CNN was calculated.

### 5.1. Synthetic Datasets

The two synthetic data sets described in Table 1 were used to perform the signal recovery using sparse recovery and deep learning. Table 2 shows the average RMSE and PSNR for each of the seven methods evaluated. ResCNN shows the smallest average RMSE for both the Gaussian and Lorentzian datasets of 0.0094 and 0.0073, respectively. Moreover, ResCNN shows the largest average PSNR of 49.0 dB for the Lorentzian dataset. For the Gaussian dataset, the sparse recovery method with Gaussian 2 shows the largest average PSNR, 49.7 dB, which is slightly higher than the 47.2 dB for ResCNN. Note that the minor difference between the two Gaussian line shape matrices results in considerable performance difference. However, reconstruction using the learned dictionaries show similar performance across all of the synthetic datasets.

**Figure 6:**
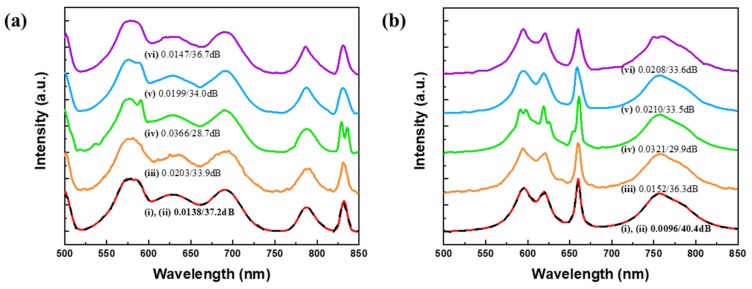
Spectral reconstructions of test spectra in synthetic datasets, (**a**) Gaussian dataset, (**b**) Lorentzian dataset. An input spectrum (solid red (i)) is compared with ResCNN (dashed black (ii)), CNN (orange (iii)), sparse recovery: Gaussian 1 (green (iv)), Gaussian 2 (blue (v)), and online dictionary learning (ODL) (purple (vi)). The baselines are shifted for clarity.

Figure 6 shows the reconstructed test spectra from each of the synthetic datasets. The solid red line (i) is the input spectra from each dataset. ResCNN is shown in dashed black line (ii), while CNN is shown in solid orange lines (iii). The reconstructed spectra using sparse recovery with Gaussian 1 (iv), Gaussian 2 (v), and ODL (vi) are shown in solid green, blue, and purple lines in respectively. Because of the similar performance from each of the learned dictionaries, only the ODL method is shown. The RMSE and PSNR of ResCNN are 0.0138 (37.2 dB) for the spectrum from the Gaussian dataset and 0.0096 (40.4 dB) for the spectrum from the Lorentzian dataset. For the selected spectra, ResCNN achieves superior reconstruction performance compared with the other four reconstructions.

Only sparse recovery with Gaussian 1 fails to recover the fine details of the input spectrum. One example of the poor ability of sparse recovery with Gaussian 1 to resolve the signal is the recovery of the peak at ~830 and 590 nm being recovered as two neighboring peaks in Figure 6a,b, respectively. CNN was unable to capture the smoothness of the spectral features compared to the other methods.

### 5.2. Noisy Synthetic Datasets

To verify the stability of ResCNN, we evaluated the accuracy of the reconstruction at various noise levels. Gaussian white noise was added to the measurement vector $n\in {\mathbb{R}}^{M\times 1}$ to Equation (2), i.e., y=Tx+n. We considered six different noise levels whose signal-to-noise ratios (SNRs) are 15, 20, 25, 30, 35, and 40 dB. The SNR (dB) is defined as $10\cdot \log_{10}\left({\left\|x\right\|}_{2}^{2}/N\sigma^{2}\right)$, where σ is the standard deviation of the noise. Using Gaussian and Lorentzian datasets, we compared the reconstruction performance of ResCNN with the sparse recovery using Gaussian 2, which shows the best reconstruction performances among sparse recovery methods in synthetic datasets. ResCNN was evaluated with the same hyper-parameters that were used for the noise-free datasets. The average RMSE and PSNR for each of the six noise levels are shown in Table 3. While ResCNN was trained using noise-free data, it outperformed the sparse recovery with Gaussian 2 at every noise level, which indicates that ResCNN remains stable even with noisy datasets. 

### 5.3. Measured Datasets

ResCNN was trained using the two measured datasets listed in Table 1, USGS and Munsell colors, and its reconstruction performance was evaluated. In addition, the signal reconstruction was performed using CNN and sparse recovery with five different sparsifying bases. Table 4 reports the average RMSE and PSNR for each of the seven methods. ResCNN achieves the smallest average RMSE and the largest average PSNR for both datasets. In the USGS dataset, the average RMSE and PSNR of ResCNN are 0.0048 and 52.4 dB, respectively. In addition, ResCNN achieves 0.0040 for the average RMSE and 50.0 dB for the average PSNR in the Munsell colors dataset. Similar to synthetic datasets, all of the learned dictionaries provided similar reconstruction performances. In addition, the small differences between Gaussian 1 and 2 show large differences in the RMSE and PSNR. The average RMSE and PSNR of the learned dictionary methods approach the values of ResCNN for Munsell colors dataset because the Munsell colors dataset has simpler spectral features than the other datasets.

**Figure 7:**
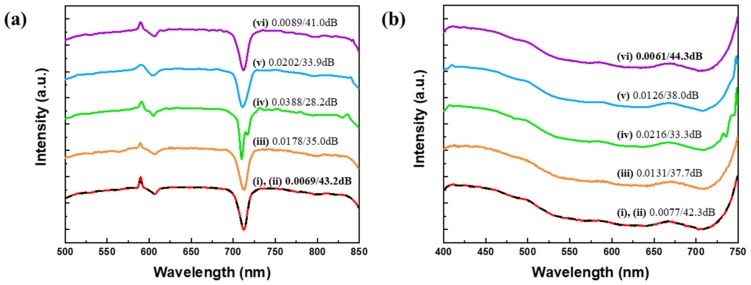
Spectral reconstructions of test spectra in measured datasets: (**a**) spectrum of organic compound dibenzothiophene in USGS dataset, (**b**) spectrum of Munsell color 5PB 2/2. The input spectrum (solid red line (i)) is compared with ResCNN (dashed black (ii)), CNN (orange (iii)), sparse recovery: Gaussian 1 (green (iv)), Gaussian 2 (blue (v)), and K-SVD (purple (vi)). The baselines are shifted for clarity.

Figure 7 shows the reconstruction results of one test spectra from each of the measured datasets. The spectrum for the organic compound dibenzothiophene in the USGS dataset is reconstructed in Figure 7a. The spectrum of Munsell color 5 PB 2/2 is shown in Figure 7b. The solid red lines are the input spectra (i). ResCNN are shown in dashed black lines (ii), and CNN are shown in solid black lines (iii). The spectra of (iv) to (vi) are reconstructed spectra using the sparse recovery with Gaussian 1, Gaussian 2, and K-SVD. Because of the best performance of the K-SVD among the learned dictionaries only the K-SVD method is shown.

The RMSE and PSNR for ResCNN are 0.0069 (43.2 dB) for the spectrum from the USGS dataset and 0.0077 (42.3 dB) for the spectrum from the Munsell colors dataset. ResCNN outperforms other approaches for the spectrum from USGS dataset. However, for the spectrum from Munsell colors dataset, the sparse recovery with K-SVD outperforms ResCNN. ResCNN achieves slightly larger RMSE and smaller PSNR.

The performances of sparse recovery with Gaussian 2 is degraded for measured datasets compared with the performance for synthetic datasets. The measured datasets have rough spectral features unlike the smooth spectral features observed in the synthetic datasets. As a result, the sparse recovery with Gaussian 2 performs worse, because of its inability to represent rough spectral features using Gaussian distribution functions. The performance of sparse recovery with dictionary learning methods are improved for measured datasets compared with the performance of synthetic datasets. Because the number of spectra in measured datasets are smaller than the number of spectra in synthetic datasets. Therefore, finding the best-fit sparsifying basis for measured datasets is easier than finding the best-fit sparsifying basis for synthetic datasets using dictionary-learning methods. Meanwhile, ResCNN shows superior reconstruction performances regardless of spectral features of datasets and the size of datasets.

## 6. Discussion

As shown in the results, we demonstrate empirically that ResCNN outperforms the sparse recovery methods and the CNN over all datasets. The sparse recovery shows unstable performance because it is highly dependent on the sparsifying basis and spectral features of dataset. This is a direct result of being unable to identify a fixed sparsifying basis that can transform any spectra into a sparse signal, which means the *a priori* structural information such as line shapes and FWHMs is required to select a consistent sparsifying basis. Learned dictionaries are used to cope with the problem of identifying a consistent sparsifying basis. The columns of learned dictionaries are composed of learned spectral features from the training dataset. While this shows an improvement in measured datasets, a learned dictionary is still limited to representing all the spectral features in the large dataset (i.e., synthetic datasets) using linear combinations of columns of the learned dictionary.

Compression approaches for summarizing information with a small number of sensors were proposed in [40]. These approaches can be exploited to generate a sparsifying basis by reducing the loss of spectral information in large datasets.

To improve the reconstruction performance in sparse recovery, pre-defined structure information and side information of unknown target signals were used in [41,42]. The reconstruction of three-dimensional electrical impedance tomography was improved by updating three-dimensional structural correlations using pre-defined structured signals [41]. To recover multi-modal data, a reconstruction framework is proposed in [42] that uses side information in unrolled optimization. Unrolled optimization approaches using deep learning were proposed in [43,44]. Deep-learning architectures were used to train hyper-parameters, such as a gradient regularizer and a step size. Using learned hyper-parameters, it was shown optimized solutions can be obtained within a fixed number of iterations. These proposed approaches for image reconstruction have assumed random sensing matrix and structured or sparse signals. In this work, however, we consider dense spectra and the sensing matrix from thin-film filters for the real implementation. Moreover, the reconstruction performance may change to a sparsifying basis as shown in results because a reconstructed spectrum $\hat{x}$ should be represented as a linear combination of columns of a fixed sparse basis Φ as $\Phi \hat{s}$.

For recovering spectra, ResCNN does not require the *a priori* knowledge of a sparsifying basis or prior information of spectral features. During training, ResCNN learns the spectral features using learnable layers, which enable it to recover the fine details for various kinds of spectra without identifying a sparsifying basis. 

**Figure 8:**
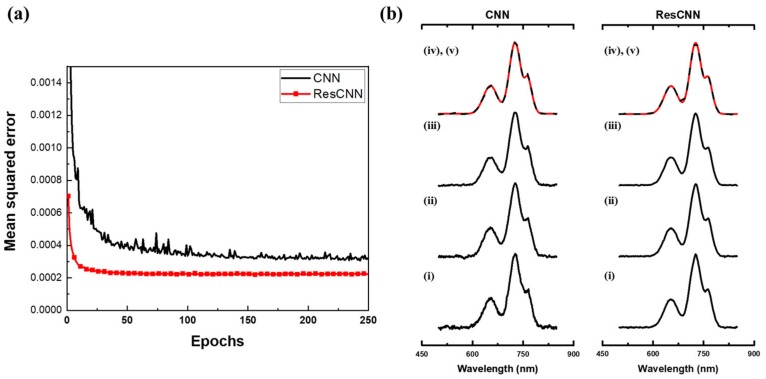
(**a**) Mean squared error of Gaussian dataset with respect to epochs. Solid black line denotes validation error of CNN, and solid red line with square symbols denotes validation error of ResCNN. (**b**) Reconstructions of a spectrum with respect to epochs where (i) to (iv) are epochs 1, 50, 150, and 250, respectively. Red line (v) denotes the original spectrum.

ResCNN is directly compared with CNN for the synthetic Gaussian dataset in Figure 8a where the mean squared error (Equation (4)) is plotted with respect to the epoch. The mean squared error for CNN and ResCNN are shown in solid black line and solid red line with square symbols, respectively. ResCNN shows a lower mean squared error than that of CNN. Moreover, ResCNN converges faster than CNN, indicating that ResCNN optimizes the learnable layers quicker, as expected based on previous research using residuals [25,29]. In contrast to the previous research that numerous residual connections were used in very deep neural networks to converge networks faster by avoiding vanishing gradient problem, we achieve spectral reconstruction improvements even with one residual connection in a moderate depth CNN.

The reconstruction of an example spectrum with respect to the number of epochs is shown in Figure 8b. Black lines ((i) to (iv)) are the reconstructed spectra at 1, 50, 150, and 250 epochs, respectively. The solid red line (v) is the original spectrum, and the series of reconstructed spectrum for ResCNN show that the reconstruction converged earlier than CNN. The increased rate of convergence is because of the residual connection in ResCNN. Overall, the reconstruction performance of ResCNN is an improvement over CNN.

Note that both ResCNN and dictionary learning for sparse recovery require a training dataset and an optimization process to learn the spectral features. While this is a time-consuming process, remember that when using a learned dictionary to recover spectra, an iterative reconstruction algorithm is required, which needs additional time and incurs a high computational cost. The benefit of ResCNN is that it gives a reconstructed spectrum immediately once the training is completed.

## 7. Conclusions

In this paper, we propose a novel ResCNN for recovering the input spectrum from the compressed measurements in CS spectroscopy. As the optical structure for CS spectroscopy, we numerically generated multilayer thin-film filters which have a small mutual coherence. Therefore, we could compressively measure input spectra with unique sensing patterns. To reconstruct the input spectra from the compressively sampled measurements, we modeled ResCNN, which has a moderate-depth of learnable layers and a residual connection. We stacked nine learnable layers: five convolutional layers and four fully-connected layers with a single residual connection between the input and output of the learnable layers. The measurements were extended by a linear transformation and then fed into ResCNN. Finally, ResCNN reconstructed the input spectra. We demonstrated the empirical reconstruction results for ResCNN using synthetic and measured datasets. We compared the reconstruction performance of ResCNN with sparse recovery using five different sparsifying bases and CNN. Compared with sparse recovery methods, ResCNN shows better reconstruction performance without the *a priori* knowledge of either a sparsifying basis or any spectral features of the spectral datasets. On the other hand, the sparse recovery methods show deviation of reconstruction performances to sparsifying bases and spectral datasets, meaning that a fixed sparsifying basis cannot represent all spectral features of input spectra. Furthermore, ResCNN shows stable reconstruction performances under noisy environments. Compared with CNN, ResCNN shows significant improvement in reconstruction performance and converges faster than CNN. In future work, we will explore compression approaches [40] and unrolled optimization approaches [43,44] for generating a sparsifying basis Φ from the training dataset to fully represent spectra without loss of spectral features.

## Figures and Tables

**Table 1:** Description of the spectral datasets.

Dataset	Training/Validation/Test	Avg. Number of Nonzero Values	Description
Gaussian dataset	8000/2000/2000	336.8/350	FWHM (nm) on the interval [2, 50], Height on the interval [0, 1]
Lorentzian dataset	8000/2000/2000	349/350	FWHM (nm) on the interval [2, 50], Height on the interval [0, 1]
US Geological Survey [32]	982/246/245	348.9/350	350–2500 nm, 2151 spectral bands (we use 350 spectral bands in 500–849 nm)
Munsell colors [33]	1066/267/267	349/350	380–780 nm, 401 spectral bands (we use 350 spectral bands in 400–749 nm)

**Table 2:** Average root mean squared errors (RMSEs) and peak signal to noise ratios (PSNRs) over synthetic datasets.

	Sparse Recovery					Deep Learning	
Dataset	Gaussian 1	Gaussian 2	K-SVD	MOD	ODL	CNN	ResCNN
Gaussian dataset	0.0226 (43.1 dB)	0.0112 (**49.7 dB**)	0.0172 (40.3 dB)	0.0174 (40.3 dB)	0.0161 (41.1 dB)	0.0132 (40.5 dB)	**0.0094** (47.2 dB)
Lorentzian dataset	0.0146 (44.9 dB)	0.0094 (47.5 dB)	0.0136 (42.3 dB)	0.0137 (42.3 dB)	0.0127 (42.9 dB)	0.0101 (42.8 dB)	**0.0073** **(49.0 dB)**

**Table 3:** Average RMSE and PSNR under various signal-to-noise ratios (SNRs, dB) with synthetic datasets.

		SNR (dB)					
**Dataset**	Method	15 dB	20 dB	25 dB	30 dB	35 dB	40 dB
**Gaussian Dataset**	Sparse recovery + Gaussian 2	0.0796 (22.7 dB)	0.0482 (27.1 dB)	0.0308 (31.2 dB)	0.0215 (34.8 dB)	0.0166 (37.9 dB)	0.0138 (40.7 dB)
	ResCNN	**0.0671 (24.2 dB)**	**0.0401 (28.7 dB)**	**0.0251 (32.9 dB)**	**0.0171 (36.6 dB)**	**0.0130 (39.8 dB)**	**0.0110 (42.4 dB)**
**Lorentzian Dataset**	Sparse recovery + Gaussian 2	0.0817 (22.6 dB)	0.0483 (27.1 dB)	0.0300 (31.2 dB)	0.0201 (35.0 dB)	0.0147 (38.5 dB)	0.0119 (41.4 dB)
	ResCNN	**0.0689 (24.1 dB)**	**0.0404 (28.7 dB)**	**0.0243 (33.1 dB)**	**0.0157 (37.1 dB)**	**0.0113 (40.6 dB)**	**0.0091 (43.4 dB)**

**Table 4:** Average RMSEs and PSNRs for the measured datasets.

	Sparse Recovery					Deep Learning	
Dataset	Gaussian 1	Gaussian 2	K-SVD	MOD	ODL	CNN	ResCNN
USGS [32]	0.0081 (45.3 dB)	0.0061 (48.4 dB)	0.0070 (48.5 dB)	0.0081 (47.4 dB)	0.0074 (47.6 dB)	0.0116 (40.8 dB)	**0.0048 (52.4 dB)**
Munsell colors [33]	0.0068 (44.6 dB)	0.0050 (47.5 dB)	**0.0040** (49.8 dB)	**0.0040** (49.9 dB)	0.0042 (49.5 dB)	0.0076 (43.0 dB)	**0.0040 (50.0 dB)**
